# Supplementary material for: SyNDI: synchronous network data integration framework
Source: BMC Bioinformatics. 2018 Nov 6;19:403. doi: 10.1186/s12859-018-2426-5 (PMC6219086; doi:10.1186/s12859-018-2426-5)
Supplement: Supplementary file 5 — Files of the example presented in Additional file 4. This additional file is a zip package that contains all files used in Additional file 4. Detailed descriptions of each of these files are presented in a README file included in this zip package. (GZ 30411 kb) [file 12859_2018_2426_MOESM5_ESM.gz › SyNDITools/Meme2Fimo/meme_4.9.0_4.tar.gz!/meme_4.9.0/doc/standards.html]

Meta-MEME standards


# Meta-MEME standards

- Command lines follow the POSIX.2 standard, but always use long
  options preceded by a double hyphen. Required arguments do not have
  flags associated with them and occur at the end of the command
  line:

  ```
           command --flag1 option1 --flag2 required1 required2
  ```
- Programs are written in C or Perl. C programs reside in the
  `src` directory and are compiled into an
  architecture-specific subdirectory of `bin`. Perl
  programs reside in the `bin` directory.
- All programs provide a brief usage statement on the command
  line when they are typed with no arguments. Detailed documentation
  associated with program `foo` is given in
  `doc/foo.html`.
